# Supplementary material for: Gut microbiota-testis axis: FMT improves systemic and testicular micro-environment to increase semen quality in type 1 diabetes
Source: Mol Med. 2022 Apr 25;28:45. doi: 10.1186/s10020-022-00473-w (PMC9036783; doi:10.1186/s10020-022-00473-w)
Supplement: Supplementary file 1 — Additional file 1: Table S1. Primary antibody information. [file 10020_2022_473_MOESM1_ESM.docx]

Primary antibody information

| **Gene symbol** | **Name** | **Cat. #** | **Predicted size** | **Source (Animal)** | **Company** |
| --- | --- | --- | --- | --- | --- |
| DDX4 (VASA) | DEAD (Asp Glu Ala Asp) box polypeptide | ab13840 | 76kDa | Rabbit (polyclonal) | Abcam |
| DAZL | DAZ like autosomal | ab34139 | 33kDa | Rabbit (polyclonal) | Abcam |
| SCP3/SYCP3 | Synaptonemal complex protein 3 | NB300-232 | 28kDa | Rabbit (polyclonal) | Novus Biologicals |
| SOX9 | SRY (sex-determining region Y)-box 9 protein | AB5535 | 65kDa | Rabbit (polyclonal) | Merck Millipore |
| TNP1(TP1) | Transition protein-1 | ab73135 |  | Rabbit (polyclonal) | Abcam |
| CREM | cAMP response element modulator | D152356 | 39kd | Rabbit (polyclonal) | Sangon Biotech (Shanghai) Co., Ltd. |
| B-myb | Myb related protein B | bs-5960R | 77kd | Rabbit (polyclonal) | Beijing Biosynthesis Biotechnology CO. |
| PIWIL1 | Piwi like protein 1 | ab94917 | 99kDa | Rabbit (polyclonal) | Abcam |
| ODF1 | Outer defense fiber 1 | Sc-390152 | 27kd | Mouse (monoclonal) | Santa Cruz Biotechnology, Inc. |
| PGK2 | Phosphoglycerate kinase 2 | D121803 | 45kDa | Rabbit (polyclonal) | Sangon Biotech (Shanghai) Co., Ltd. |
| Ki67 | Antigen identified by monoclonal antibody Ki 67 | bs-2130R | 358kd | Rabbit (polyclonal) | Beijing Biosynthesis Biotechnology CO. |
| Bcl-2 | B cell lymphoma 2 | bs-4563R | 26kDa | Rabbit (polyclonal) | Beijing Biosynthesis Biotechnology CO. |
| CCR7 | CCR7 | bs-1305R | 42kDa | Rabbit | Beijing Biosynthesis Biotechnology CO. |
| CCL21 | CCL21 | bs-1666R | 15kDa | Rabbit | Beijing Biosynthesis Biotechnology CO. |
| CD163 | CD163 | ab213612 | 121 kDa | Rabbit | Abcam |
| P53 | Transformation related protein 53 | bs-8687R | 53kd | Rabbit (polyclonal) | Beijing Biosynthesis Biotechnology CO. |
| Bax | BCL2-Associated X | bs-4564R | 21kd | Rabbit (polyclonal) | Beijing Biosynthesis Biotechnology CO. |
| Bcl-xl | Bcl-xl | bs-1336R | 26kd | Rabbit (polyclonal) | Beijing Biosynthesis Biotechnology CO. |
| SOX9 | SRY (sex-determining region Y)-box 9 protein | AB5535 | 65kDa | Rabbit (polyclonal) | Merck Millipore |
| PLZF1 | **promyelocytic leukaemia zinc finger** | Ab39354 | 72kDa | Rabbit (polyclonal) | Abcam |
| actin | actin | Ab3280 | 42kDa | Rabbit (polyclonal) | Abcam |
