# Supplementary figures and images for: Gut microbiota-testis axis: FMT improves systemic and testicular micro-environment to increase semen quality in type 1 diabetes
Source: Mol Med. 2022 Apr 25;28:45. doi: 10.1186/s10020-022-00473-w (PMC9036783; doi:10.1186/s10020-022-00473-w)

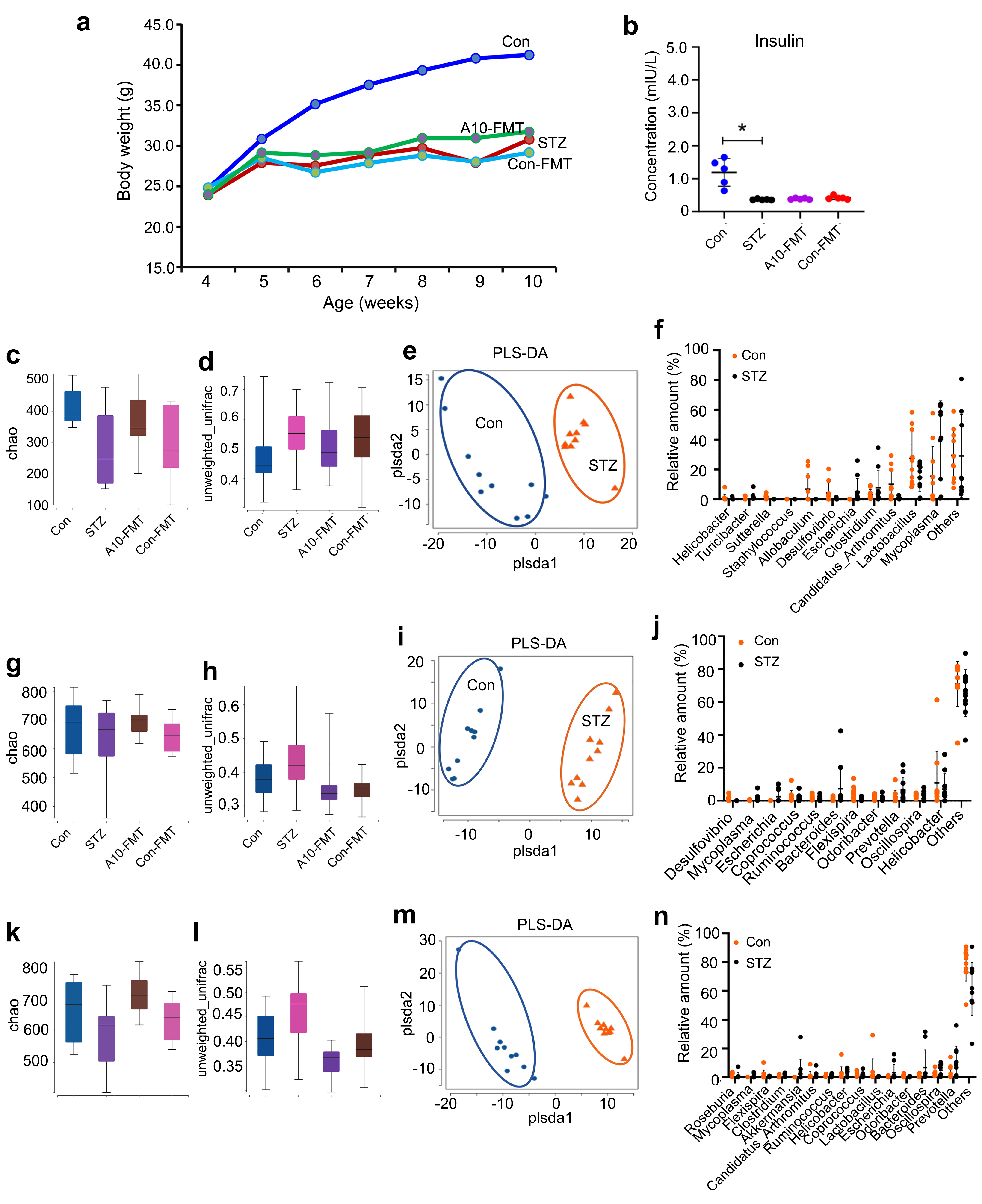

Supplement: Supplementary file 2 — Additional file 2: Fig. S1. Body weight and gut microbiota changes (STZ vs. Con). a Animal bodyweight. The y-axis represents the body weight (g). The x-axis represents the age (weeks). b Blood insulin levels. The y-axis represents the concentration (mIU/L). The x-axis represents the treatment. c The alpha index of the small intestine microbiota (Chao index). The y-axis represents the relative amount. The x-axis represents the treatment. d The beta index of small intestinal microbiota. The y-axis represents the relative amount. The x-axis represents the treatment. e PLS-DA (OTU) of small intestine microbiota in STZ and Con groups. f Small intestine microbiota levels at the genus level in STZ and Con groups. The y-axis represents the relative amount (%). The x-axis represents the individual microbiota. g The alpha index of the cecum microbiota (Chao index). The y-axis represents the relative amount. The x-axis represents the treatment. h The beta index of cecum microbiota. The y-axis represents the relative amount. The x-axis represents the treatment. i PLS-DA (OTU) of cecum microbiota in STZ and Con groups. j Cecum microbiota levels at the genus level in STZ and Con groups. The y-axis represents the relative amount (%). The x-axis represents the individual microbiota. k The alpha index of the colon microbiota (Chao index). The y-axis represents the relative amount. The x-axis represents the treatment. l The beta index of colon microbiota. The y-axis represents the relative amount. The x-axis represents the treatment. m PLS-DA (OTU) of colon microbiota in STZ and Con groups. n Colon microbiota levels at the genus level in STZ and Con groups. The y-axis represents the relative amount (%). The x-axis represents the individual microbiota. Fig. S2. a PLS-DA (OTU) of cecum microbiota in STZ, A10-FMT, and Con-FMT groups. b Cecum microbiota levels at the genus level in STZ, A10-FMT, and Con-FMT groups. The y-axis represents the relative amount (%). The x-axis represents the [file 10020_2022_473_MOESM2_ESM.zip › Fig. S1R.tif]

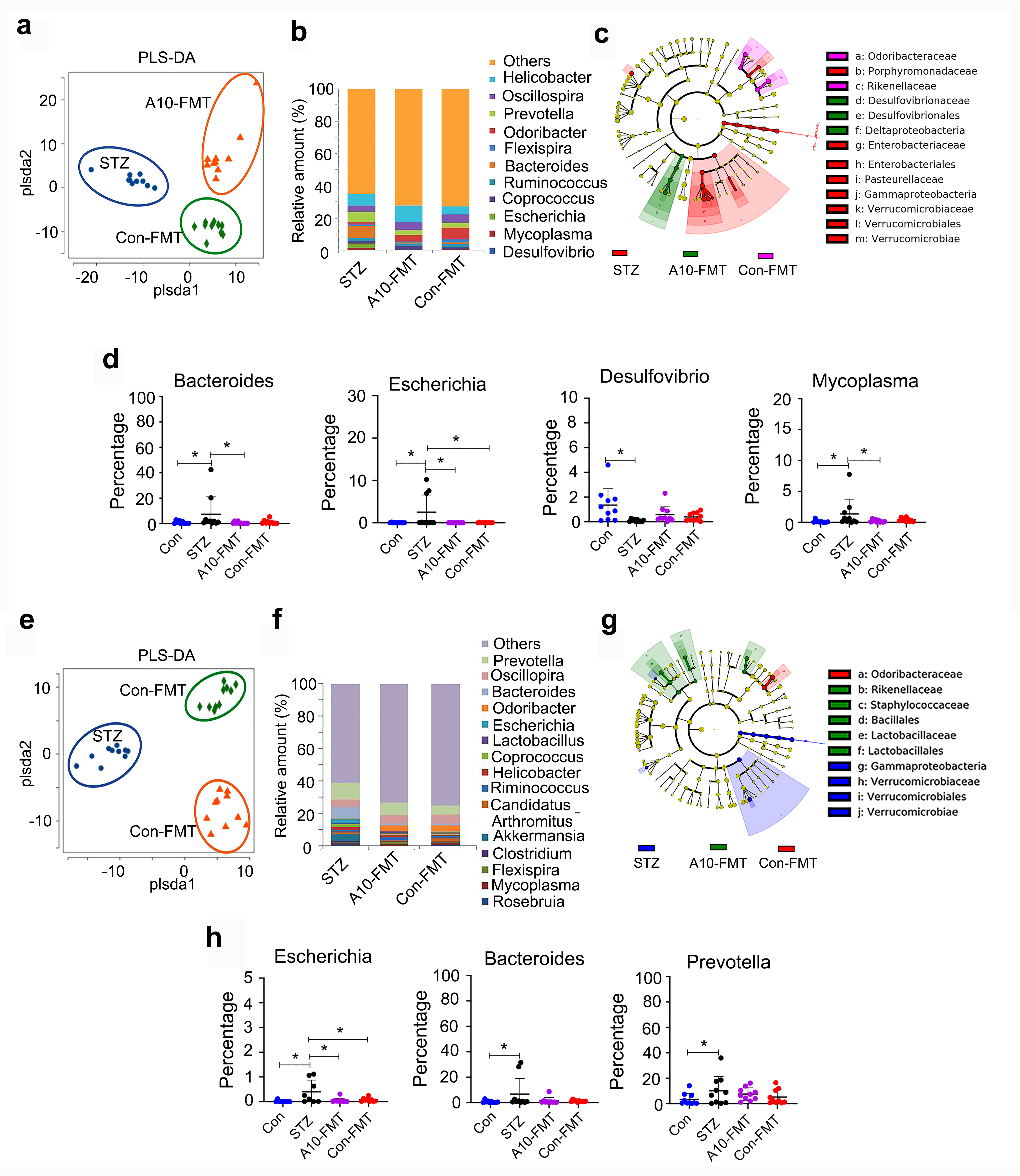

Supplement: Supplementary file 2 — Additional file 2: Fig. S1. Body weight and gut microbiota changes (STZ vs. Con). a Animal bodyweight. The y-axis represents the body weight (g). The x-axis represents the age (weeks). b Blood insulin levels. The y-axis represents the concentration (mIU/L). The x-axis represents the treatment. c The alpha index of the small intestine microbiota (Chao index). The y-axis represents the relative amount. The x-axis represents the treatment. d The beta index of small intestinal microbiota. The y-axis represents the relative amount. The x-axis represents the treatment. e PLS-DA (OTU) of small intestine microbiota in STZ and Con groups. f Small intestine microbiota levels at the genus level in STZ and Con groups. The y-axis represents the relative amount (%). The x-axis represents the individual microbiota. g The alpha index of the cecum microbiota (Chao index). The y-axis represents the relative amount. The x-axis represents the treatment. h The beta index of cecum microbiota. The y-axis represents the relative amount. The x-axis represents the treatment. i PLS-DA (OTU) of cecum microbiota in STZ and Con groups. j Cecum microbiota levels at the genus level in STZ and Con groups. The y-axis represents the relative amount (%). The x-axis represents the individual microbiota. k The alpha index of the colon microbiota (Chao index). The y-axis represents the relative amount. The x-axis represents the treatment. l The beta index of colon microbiota. The y-axis represents the relative amount. The x-axis represents the treatment. m PLS-DA (OTU) of colon microbiota in STZ and Con groups. n Colon microbiota levels at the genus level in STZ and Con groups. The y-axis represents the relative amount (%). The x-axis represents the individual microbiota. Fig. S2. a PLS-DA (OTU) of cecum microbiota in STZ, A10-FMT, and Con-FMT groups. b Cecum microbiota levels at the genus level in STZ, A10-FMT, and Con-FMT groups. The y-axis represents the relative amount (%). The x-axis represents the [file 10020_2022_473_MOESM2_ESM.zip › Fig. S2R.tif]

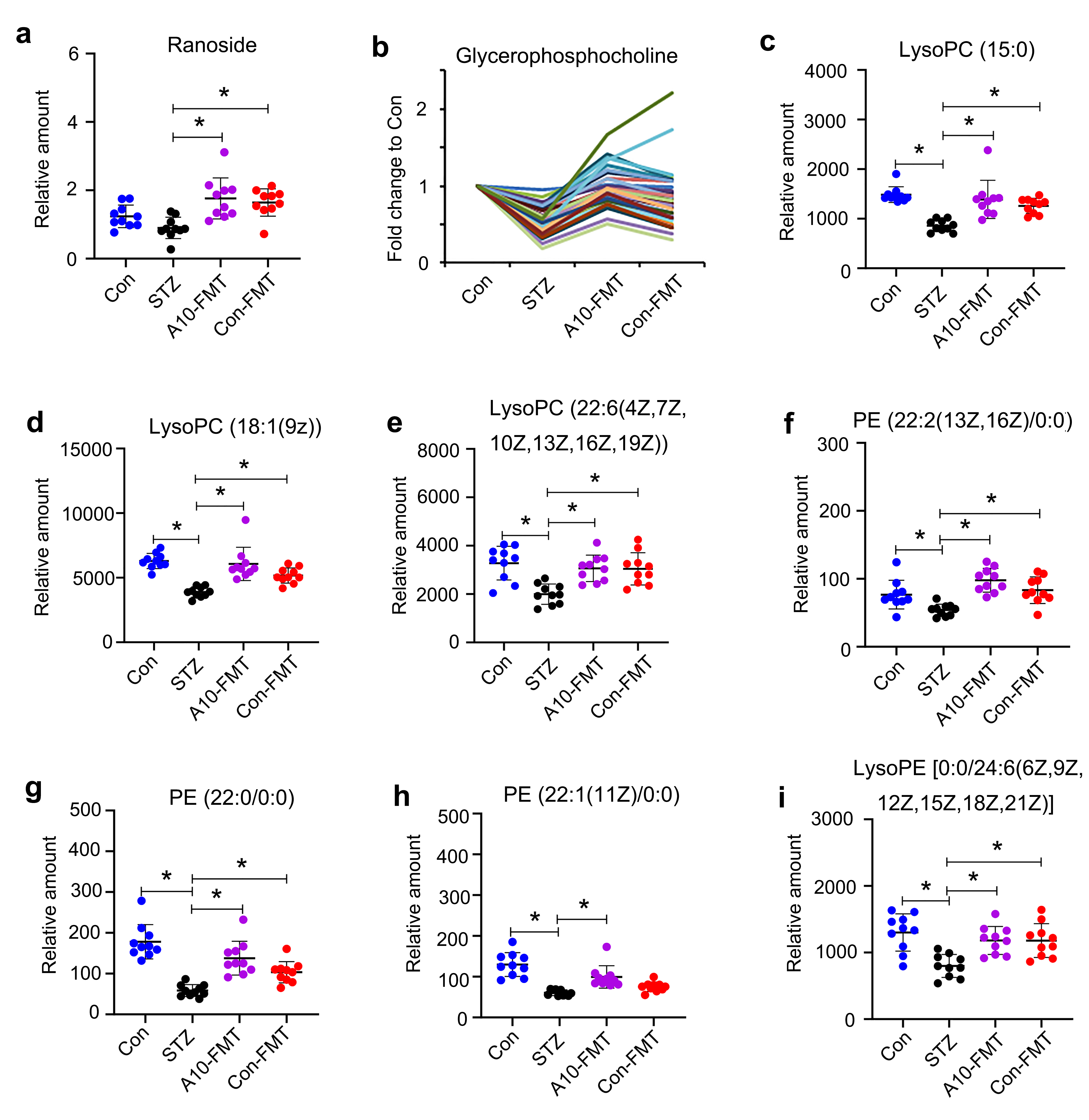

Supplement: Supplementary file 2 — Additional file 2: Fig. S1. Body weight and gut microbiota changes (STZ vs. Con). a Animal bodyweight. The y-axis represents the body weight (g). The x-axis represents the age (weeks). b Blood insulin levels. The y-axis represents the concentration (mIU/L). The x-axis represents the treatment. c The alpha index of the small intestine microbiota (Chao index). The y-axis represents the relative amount. The x-axis represents the treatment. d The beta index of small intestinal microbiota. The y-axis represents the relative amount. The x-axis represents the treatment. e PLS-DA (OTU) of small intestine microbiota in STZ and Con groups. f Small intestine microbiota levels at the genus level in STZ and Con groups. The y-axis represents the relative amount (%). The x-axis represents the individual microbiota. g The alpha index of the cecum microbiota (Chao index). The y-axis represents the relative amount. The x-axis represents the treatment. h The beta index of cecum microbiota. The y-axis represents the relative amount. The x-axis represents the treatment. i PLS-DA (OTU) of cecum microbiota in STZ and Con groups. j Cecum microbiota levels at the genus level in STZ and Con groups. The y-axis represents the relative amount (%). The x-axis represents the individual microbiota. k The alpha index of the colon microbiota (Chao index). The y-axis represents the relative amount. The x-axis represents the treatment. l The beta index of colon microbiota. The y-axis represents the relative amount. The x-axis represents the treatment. m PLS-DA (OTU) of colon microbiota in STZ and Con groups. n Colon microbiota levels at the genus level in STZ and Con groups. The y-axis represents the relative amount (%). The x-axis represents the individual microbiota. Fig. S2. a PLS-DA (OTU) of cecum microbiota in STZ, A10-FMT, and Con-FMT groups. b Cecum microbiota levels at the genus level in STZ, A10-FMT, and Con-FMT groups. The y-axis represents the relative amount (%). The x-axis represents the [file 10020_2022_473_MOESM2_ESM.zip › Fig. S3R.tif]

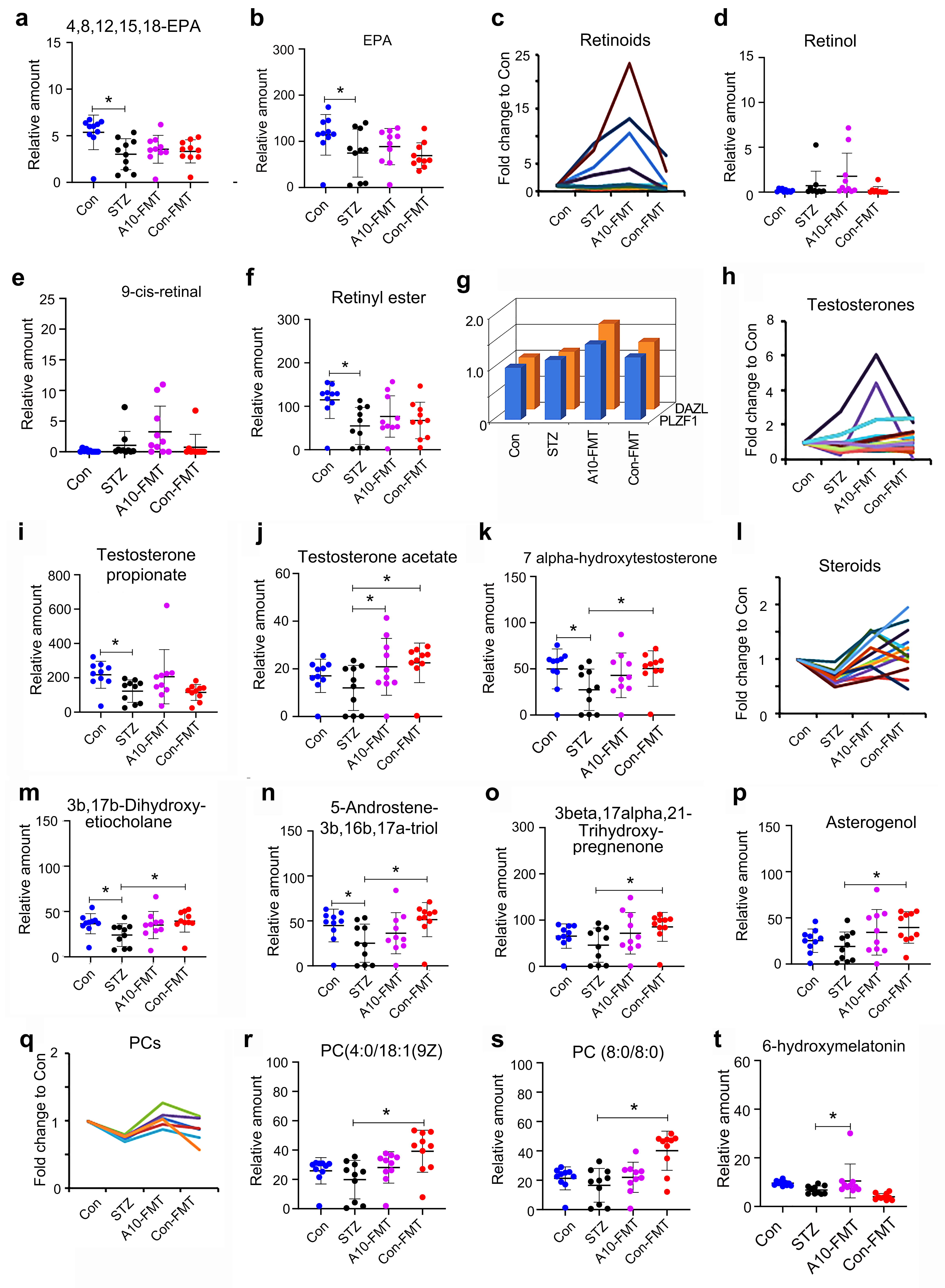

Supplement: Supplementary file 2 — Additional file 2: Fig. S1. Body weight and gut microbiota changes (STZ vs. Con). a Animal bodyweight. The y-axis represents the body weight (g). The x-axis represents the age (weeks). b Blood insulin levels. The y-axis represents the concentration (mIU/L). The x-axis represents the treatment. c The alpha index of the small intestine microbiota (Chao index). The y-axis represents the relative amount. The x-axis represents the treatment. d The beta index of small intestinal microbiota. The y-axis represents the relative amount. The x-axis represents the treatment. e PLS-DA (OTU) of small intestine microbiota in STZ and Con groups. f Small intestine microbiota levels at the genus level in STZ and Con groups. The y-axis represents the relative amount (%). The x-axis represents the individual microbiota. g The alpha index of the cecum microbiota (Chao index). The y-axis represents the relative amount. The x-axis represents the treatment. h The beta index of cecum microbiota. The y-axis represents the relative amount. The x-axis represents the treatment. i PLS-DA (OTU) of cecum microbiota in STZ and Con groups. j Cecum microbiota levels at the genus level in STZ and Con groups. The y-axis represents the relative amount (%). The x-axis represents the individual microbiota. k The alpha index of the colon microbiota (Chao index). The y-axis represents the relative amount. The x-axis represents the treatment. l The beta index of colon microbiota. The y-axis represents the relative amount. The x-axis represents the treatment. m PLS-DA (OTU) of colon microbiota in STZ and Con groups. n Colon microbiota levels at the genus level in STZ and Con groups. The y-axis represents the relative amount (%). The x-axis represents the individual microbiota. Fig. S2. a PLS-DA (OTU) of cecum microbiota in STZ, A10-FMT, and Con-FMT groups. b Cecum microbiota levels at the genus level in STZ, A10-FMT, and Con-FMT groups. The y-axis represents the relative amount (%). The x-axis represents the [file 10020_2022_473_MOESM2_ESM.zip › Fig. S4R.tif]

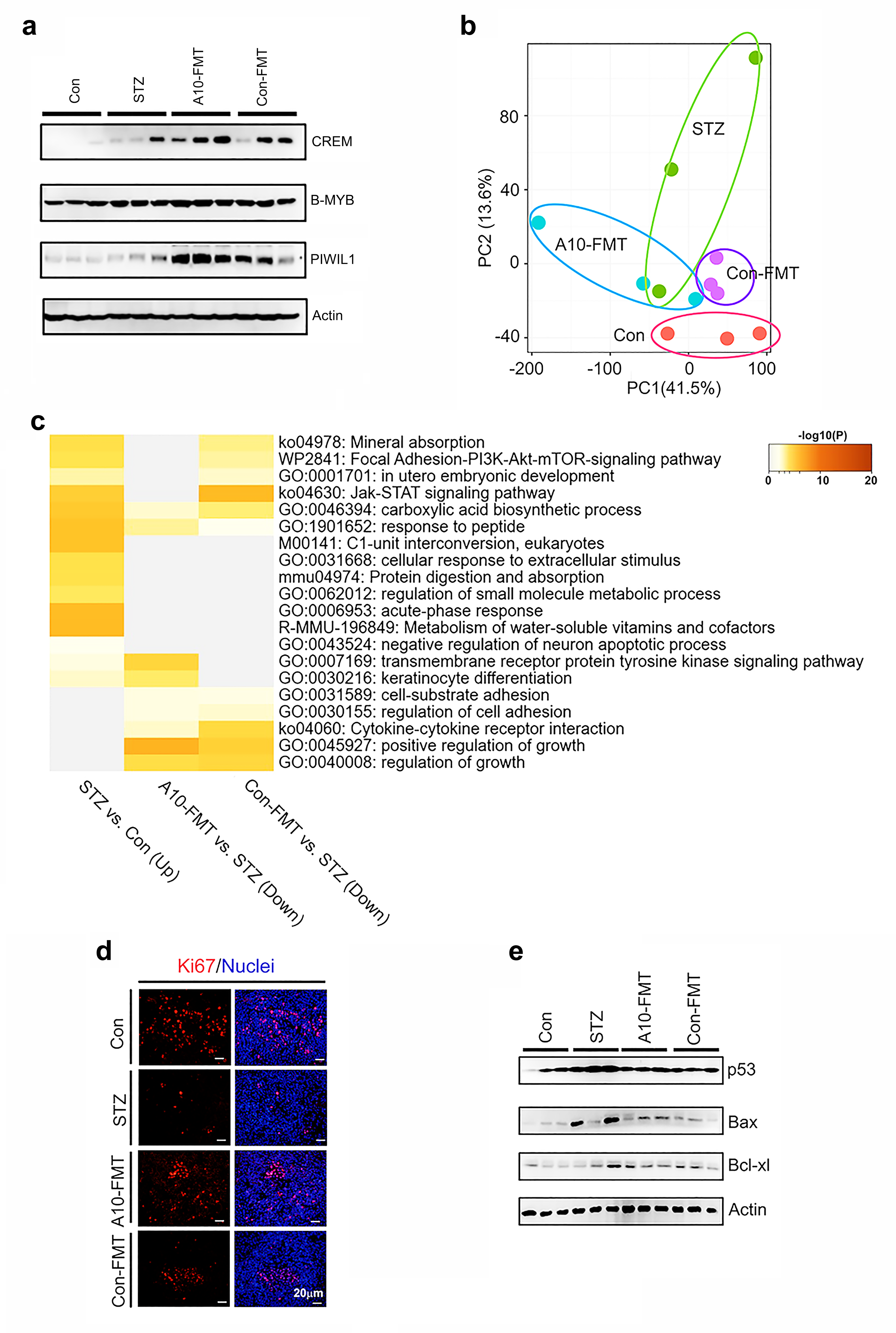

Supplement: Supplementary file 2 — Additional file 2: Fig. S1. Body weight and gut microbiota changes (STZ vs. Con). a Animal bodyweight. The y-axis represents the body weight (g). The x-axis represents the age (weeks). b Blood insulin levels. The y-axis represents the concentration (mIU/L). The x-axis represents the treatment. c The alpha index of the small intestine microbiota (Chao index). The y-axis represents the relative amount. The x-axis represents the treatment. d The beta index of small intestinal microbiota. The y-axis represents the relative amount. The x-axis represents the treatment. e PLS-DA (OTU) of small intestine microbiota in STZ and Con groups. f Small intestine microbiota levels at the genus level in STZ and Con groups. The y-axis represents the relative amount (%). The x-axis represents the individual microbiota. g The alpha index of the cecum microbiota (Chao index). The y-axis represents the relative amount. The x-axis represents the treatment. h The beta index of cecum microbiota. The y-axis represents the relative amount. The x-axis represents the treatment. i PLS-DA (OTU) of cecum microbiota in STZ and Con groups. j Cecum microbiota levels at the genus level in STZ and Con groups. The y-axis represents the relative amount (%). The x-axis represents the individual microbiota. k The alpha index of the colon microbiota (Chao index). The y-axis represents the relative amount. The x-axis represents the treatment. l The beta index of colon microbiota. The y-axis represents the relative amount. The x-axis represents the treatment. m PLS-DA (OTU) of colon microbiota in STZ and Con groups. n Colon microbiota levels at the genus level in STZ and Con groups. The y-axis represents the relative amount (%). The x-axis represents the individual microbiota. Fig. S2. a PLS-DA (OTU) of cecum microbiota in STZ, A10-FMT, and Con-FMT groups. b Cecum microbiota levels at the genus level in STZ, A10-FMT, and Con-FMT groups. The y-axis represents the relative amount (%). The x-axis represents the [file 10020_2022_473_MOESM2_ESM.zip › Fig. S5R.tif]

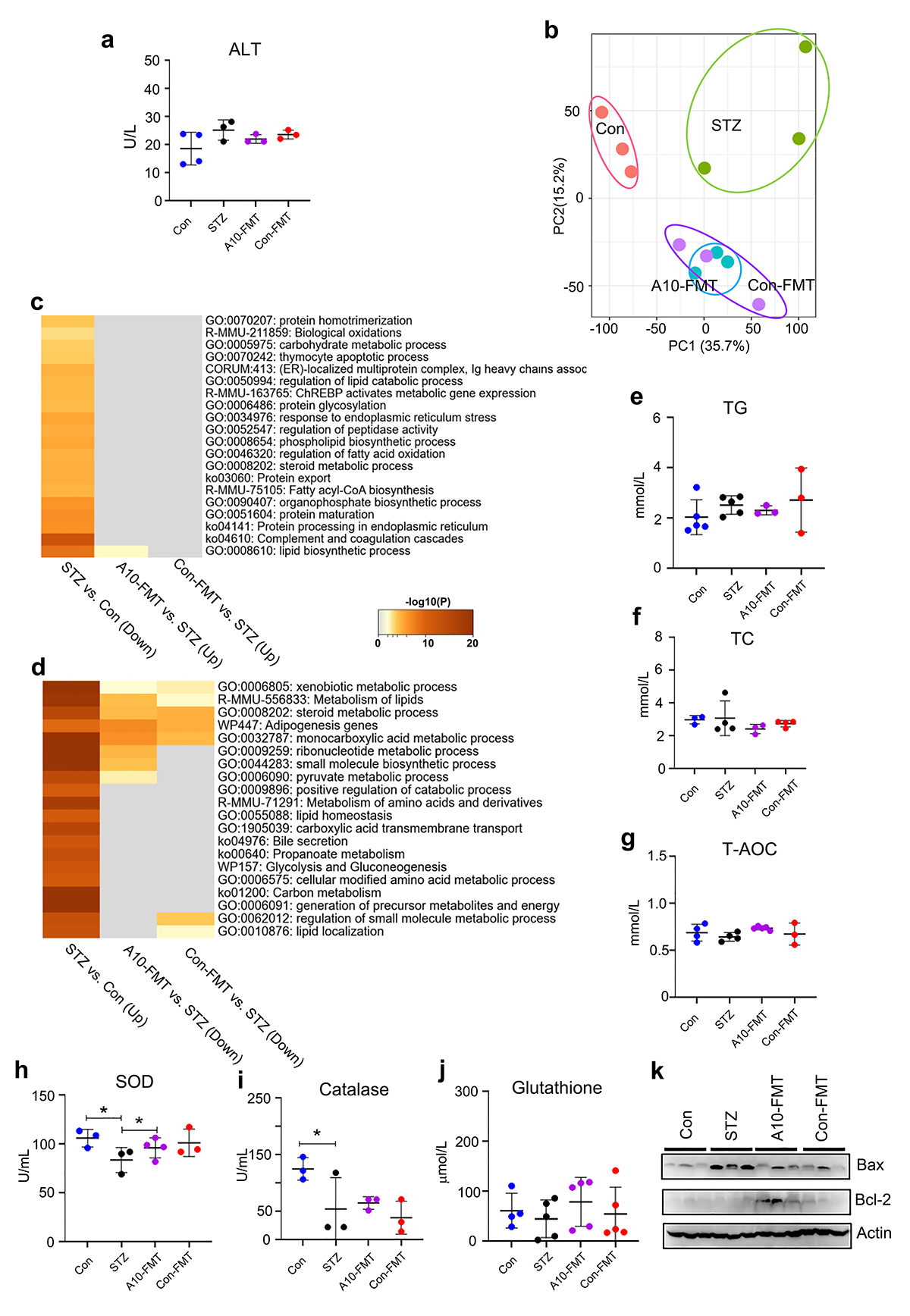

Supplement: Supplementary file 2 — Additional file 2: Fig. S1. Body weight and gut microbiota changes (STZ vs. Con). a Animal bodyweight. The y-axis represents the body weight (g). The x-axis represents the age (weeks). b Blood insulin levels. The y-axis represents the concentration (mIU/L). The x-axis represents the treatment. c The alpha index of the small intestine microbiota (Chao index). The y-axis represents the relative amount. The x-axis represents the treatment. d The beta index of small intestinal microbiota. The y-axis represents the relative amount. The x-axis represents the treatment. e PLS-DA (OTU) of small intestine microbiota in STZ and Con groups. f Small intestine microbiota levels at the genus level in STZ and Con groups. The y-axis represents the relative amount (%). The x-axis represents the individual microbiota. g The alpha index of the cecum microbiota (Chao index). The y-axis represents the relative amount. The x-axis represents the treatment. h The beta index of cecum microbiota. The y-axis represents the relative amount. The x-axis represents the treatment. i PLS-DA (OTU) of cecum microbiota in STZ and Con groups. j Cecum microbiota levels at the genus level in STZ and Con groups. The y-axis represents the relative amount (%). The x-axis represents the individual microbiota. k The alpha index of the colon microbiota (Chao index). The y-axis represents the relative amount. The x-axis represents the treatment. l The beta index of colon microbiota. The y-axis represents the relative amount. The x-axis represents the treatment. m PLS-DA (OTU) of colon microbiota in STZ and Con groups. n Colon microbiota levels at the genus level in STZ and Con groups. The y-axis represents the relative amount (%). The x-axis represents the individual microbiota. Fig. S2. a PLS-DA (OTU) of cecum microbiota in STZ, A10-FMT, and Con-FMT groups. b Cecum microbiota levels at the genus level in STZ, A10-FMT, and Con-FMT groups. The y-axis represents the relative amount (%). The x-axis represents the [file 10020_2022_473_MOESM2_ESM.zip › Fig. S6R.tif]
